# Supplementary material for: ToxiVerse: A Public Platform for Chemical Toxicity Data Sharing and Customizable Predictive Modeling
Source: bioRxiv. 2026 Mar 2:2026.02.26.708255. Preprint. [Version 1] doi: 10.64898/2026.02.26.708255 (PMC13001432; doi:10.64898/2026.02.26.708255)
Supplement: Supplement 2 — Supplementary Figure S1. The Bioprofiler functions overview: generating five distinct outputs, each available for download with sample outputs provided. (A) Heatmap representation of initial bioprofile. (B) Box plot of performance metrics for models built on the selected assays. (C) The initial bioprofile shown as chemical-bioactivity matrix with a tabular format of compound activity outcomes (−1 = inactive, 0 = inconclusive, 1 = active). (D) The performance metrics file containing evaluation results for the models based on selected assays. (E) A complete bioprofile with filled data gaps. A represents Assay and C represents chemical in the figure. [file media-2.pdf]

A

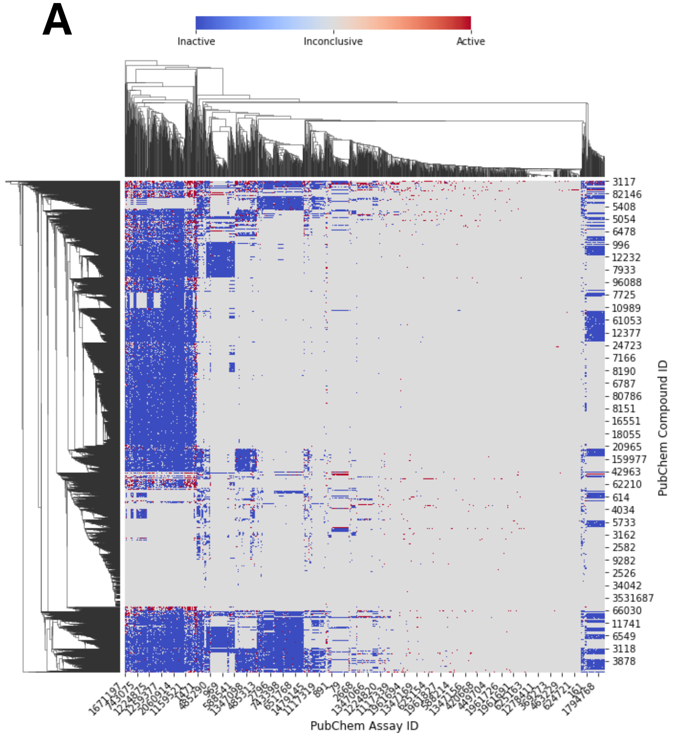

Heatmap

B

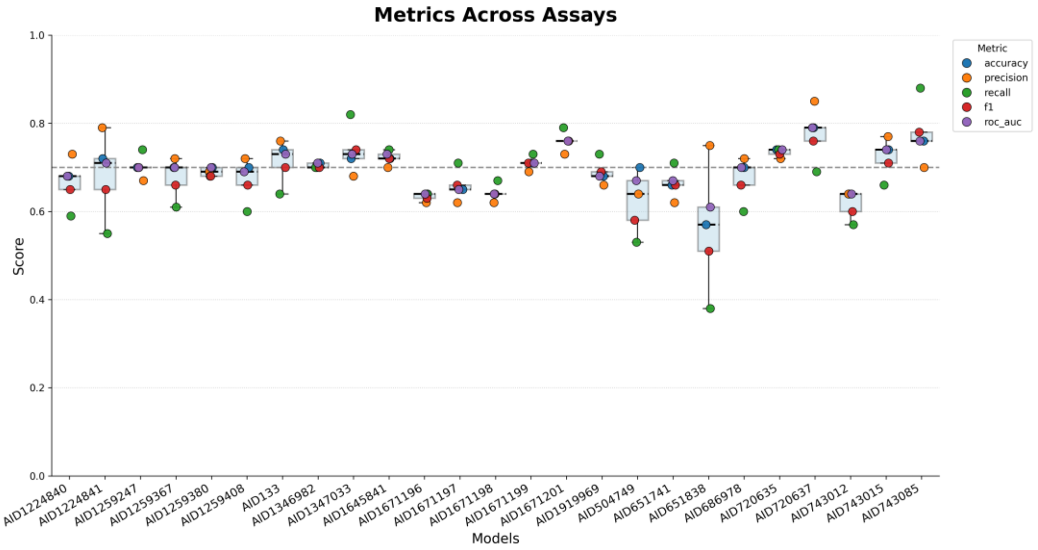

Metrics Plot

C

Initial Bioprofile

CID	A1	A2	A3	A4	...	An
C1	1	-1	0	0	...	-1
C2	-1	0	0	-1	...	0
C3	0	0	0	0	...	0
C4	1	0	-1	0	...	1
...	...	...	...	...	...	...
Cn	-1	0	0	0	...	0

D

Model Metrics

Model	accuracy	precision	recall	f1	roc_auc
A1	0.68	0.73	0.59	0.65	0.68
A2	0.72	0.79	0.55	0.65	0.71
A3	0.7	0.68	0.7	0.69	0.7
A4	0.7	0.67	0.74	0.7	0.7
...	...	...	...	...	...
A25	0.7	0.72	0.61	0.66	0.7

E

Complete Bioprofile

CID	A1	A2	A3	A4	...	An
C1	1	-1	-1	1	...	-1
C2	-1	-1	-1	-1	...	-1
C3	-1	1	-1	-1	...	-1
C4	1	-1	-1	-1	...	1
...	...	...	...	...	...	...
Cn	-1	1	1	-1	...	-1
