# Supplementary figures and images for: ToxiVerse: A Public Platform for Chemical Toxicity Data Sharing and Customizable Predictive Modeling
Source: bioRxiv. 2026 Mar 2:2026.02.26.708255. Preprint. [Version 1] doi: 10.64898/2026.02.26.708255 (PMC13001432; doi:10.64898/2026.02.26.708255)

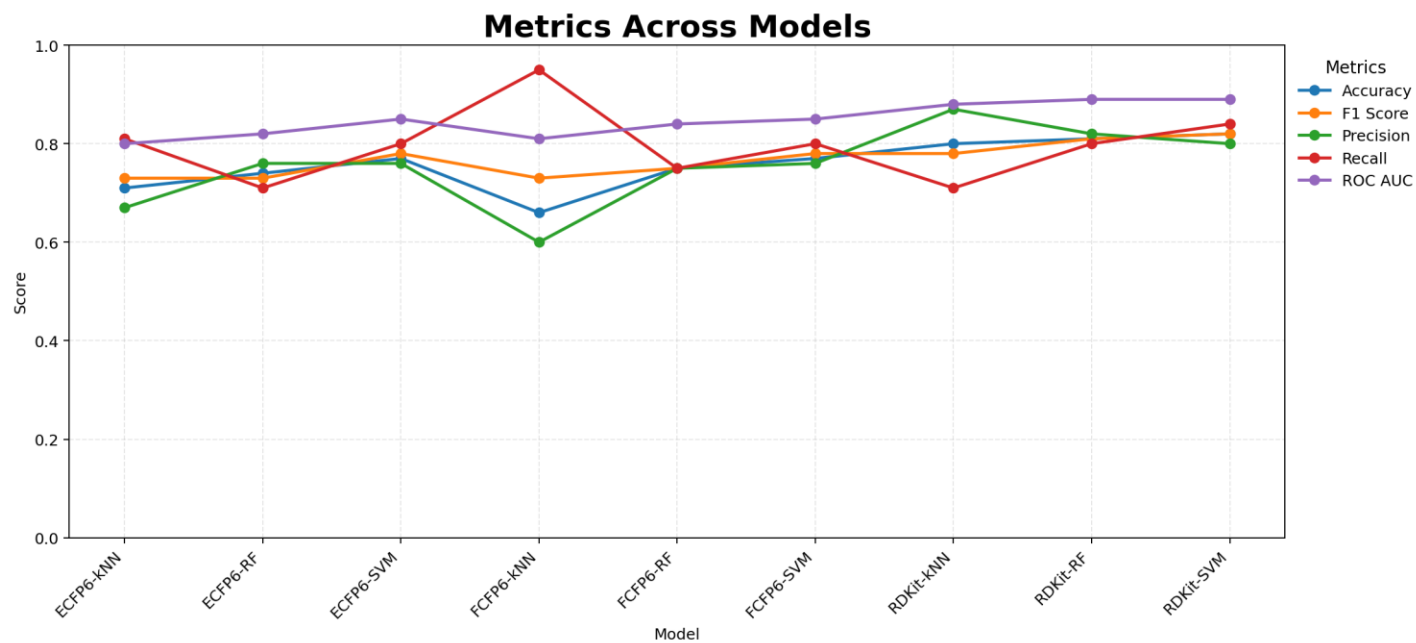

Supplement: Supplement 3 — Supplementary Figure S2. Performance metrics of the classification models for the estrogen receptor dataset. [file media-3.pdf]
